# Immune Response to COVID-19 Vaccination in Frontline Healthcare Workers

**DOI:** 10.3390/vaccines12020199

**Published:** 2024-02-15

**Authors:** Birute Strukcinskiene, Zydre Valotkiene, Jonas Jurgaitis, Rasa Grigoliene, Agnieszka Genowska

**Affiliations:** 1 Faculty of Health Sciences, Klaipeda University, LT-92294 Klaipeda, Lithuania; zydre.valotkiene@santa.lt (Z.V.); jonas.jurgaitis@ku.lt (J.J.); 2 Epidemiology Sub-Division, Infection Control Department, Vilnius University Hospital Santaros Klinikos, LT-08661 Vilnius, Lithuania; 3 Faculty of Marine Technologies and Natural Sciences, Klaipeda University, LT-92294 Klaipeda, Lithuania; rasa.grigoliene@ku.lt; 4 Department of Public Health, Medical University of Bialystok, 15-295 Bialystok, Poland

**Keywords:** vaccination, immune response, antibodies, COVID-19 management, healthcare sector, frontline workers

## Abstract

This study evaluated the immune response to vaccination against COVID-19 in 534 healthcare frontline workers in Vilnius, Lithuania. The incidence of COVID-19 was reduced significantly after vaccination started in the healthcare sector. SARS-CoV-2 antibodies were detected in groups V–VII and this level of antibodies was found to be effective in preventing COVID-19. Sustained immune response was achieved after two vaccination doses, which remained stable for up to 6 months. After the booster dose, antibody levels remained high for an additional 12 months. Although SARS-CoV-2 antibody levels decreased after 6 months, even lower levels of antibodies provided protection against the Delta strain. The booster dose distributed the antibody titer in the high-level antibody groups, offering maximum protection at 12 months. However, even individuals with high antibody titers were observed to contract COVID-19 after vaccination with a booster dose and 6 months in the presence of the Omicron strain. Unfortunately, high levels of antibodies did not provide protection against the new strain of COVID-19 (the Omicron variant), posing a risk of infection. When comparing the antibody titer of vaccinated participants without COVID-19 and those with COVID-19, the change in antibodies after vaccination was significantly lower in infected participants. Individuals with comorbidities and specific conditions had lower antibody levels.

## 1. Introduction

Immunization is an effective tool for infectious disease management and the protection of human lives worldwide. Vaccination is an essential measure in public health strategies for the prevention of communicable diseases. The WHO declares that it is currently possible to vaccinate against more than 20 life-threatening diseases. Vaccination creates protection with the body’s natural defenses, and society lives longer and healthier lives. Today, up to 5 million lives are saved each year by vaccination against diseases such as diphtheria, tetanus, pertussis, influenza, and measles [1].

Recent COVID-19 outbreaks have revealed a new attitude to vaccination in order to control pandemic outbreaks. Vaccination on a large scale provides an opportunity to avoid getting a COVID-19 infection. All EU-licensed vaccines have been shown to prevent hospitalization and severe disease and reduce the risk of death, with a population-wide effectiveness of more than 80% [2]. The European Commission is paying more attention to the volume of vaccine supply, as well as the development of new vaccines for stopping the shedding of viruses of various strains.

### 1.1. Immunization of Healthcare Workers

Studies on COVID-19 revealed that healthcare workers are at a greater risk of infection due to dangerous working conditions. Healthcare workers had a 2.9 times higher incidence risk ratio for getting a COVID-19 infection than the general population. Frontline healthcare employees who worked with COVID-19 patients were infected more often, representing one of the most vulnerable groups [3,4,5,6].

Due to the extremely fast spread of the dangerous virus and the transmission of the asymptomatic virus, the greatest risk of contracting COVID-19 lies with hospital staff and healthcare workers, because they are in close contact with patients. Therefore, the strictest prevention and control measures were applied to healthcare staff in order to protect them, and also the patients. Healthcare workers must frequently wash and disinfect their hands and use antiseptics and other personal protective equipment, such as masks or respirators, glasses, and visors. In addition, vaccinations are recommended and encouraged. Therefore, nowadays, when COVID-19 has become a constant companion in our daily lives, vaccination is an irreplaceable means of preventing this epidemic [7]. Studies revealed the effectiveness of vaccination against COVID-19 and also after getting infected with SARS-CoV-2 in healthcare employees [8].

### 1.2. Immune Response to COVID-19 Vaccination

Substantial immune memory is generated involving four major types of immune memory for approximately 6 months after COVID-19 in almost all (95%) infected persons. Each type of immune memory (antibodies, memory B, CD4^+^ T, and CD8^+^ T cells) has a protective, but different, response [9,10]. Nevertheless, the immune response is poorer, with a weaker adaptive immune response to SARS-CoV-2 and as the individual gets older [10]. In vaccinated individuals, high levels of antibodies were observed after the first dose of the vaccine against COVID-19. A study on the levels of SARS-CoV-2 antibodies among people vaccinated with three different vaccines, with the full vaccination schedule (two doses) and a booster dose, found high seropositivity and high antibody titers after vaccination [11]. Various international studies confirmed that a booster dose could significantly increase the immunity response as well [11,12,13,14,15]. Long-term effects of vaccines on antibody levels showed that the decrease in antibody titer was significantly associated with the male sex, older age, and time after vaccination [11]. Vaccination in young people induces more effective, faster, and longer antibody production compared with older people [8,16]. A lower number of lymphocytes in the blood and a higher body mass index lead to a lower immune response. In women, due to greater hormonal changes, seroconversion occurs faster than in men, but they quickly become similar [16].

Scientific studies have revealed increasing evidence that vaccines produce a more consistent and higher-titer primary immune response after receiving a full vaccination than after being infected with COVID-19 [17,18,19]. Therefore, vaccination is the main and indisputable way to fight infection. Revaccination is the most effective mean of stimulating the production of new antibodies and activating old antibodies. This is the only way to prevent the spread of the disease in society in all age groups. After the second dose, the antibody titer increases several dozen times, and, after the third and fourth dose, the strength of the immune response is increased [20].

### 1.3. Influence of SARS-CoV-2 Strains on Antibody Production

Various strains of the SARS-CoV-2 virus have been identified in the world, but only some of them are of concern because of their ability to persist and cause many negative consequences for those infected and cause many deaths. They are as follows:

- Alpha (B.1.1.7): The first strain of concern described in the United Kingdom (UK) in December 2020;
- Beta (B.1.351): First reported in South Africa in December 2020;
- Gamma (P.1): First reported in Brazil in 2021, at the beginning of January;
- Delta (B.1.617.2): First reported in India in December 2020;
- Omicron (B.1.1.529): First reported in South Africa in November 2021 [21].

The first strain (the strongest and most deadly) of the COVID-19 infection induced a robust and long-lasting SARS-CoV-2 antibody response. The study of the blood serum of healthcare workers infected during the first wave, and during other waves (and strains), found that neutralizing antibodies remained even up to 10 months after infection. In addition, when encountering new strains, the human immune system reacted, and, due to the cross-effect of the strains, the production of antibodies continued [22,23]. When other mutated strains, such as Omicron, were exposed, an obvious weakening of immunity was observed. However, the probability of infection depends not only on the quantity but also on the quality of the strains presented at that time [20]. Currently, it is already known that the effect of the vaccine on immunity increases with each dose, but the possibility of reinfection still remains as new strains of the virus appear. Studies on the importance of vaccination and resistance to new types of Omicron found that unvaccinated people fell ill much earlier than vaccinated people [24]. The risk of reinfection with SARS-CoV-2 has been shown to increase more than 250 days after vaccination. However, even though antibody titers are lower, protection against serious illness or death remains. In the presence of various strains of the virus, the reaction to infection and antibody production could be different. In addition, antibodies are mostly activated when exposed to primary variations of the SARS-CoV-2 virus strain [25].

### 1.4. Comorbidities with Specific Conditions and Coronavirus Disease (COVID-19)

All healthy unvaccinated individuals exposed to any strain of the SARS-CoV-2 virus can become infected and ill depending on their body’s resistance. For individuals who often have other comorbidities, immunosuppressive conditions, falling ill with COVID-19 can be disastrous [7]. Based on scientific research, it is stated that a person’s comorbidities and specific conditions are of great importance and could determine the severity of the disease. It was found that cardiovascular diseases (32%), diabetes (30%), and chronic lung disease (18%) have the greatest influence on the course and outcome of the disease, while obesity, chronic, oncological diseases, and transplantation complicate the course of the disease and often worsen the outcome [21,26].

Studies on vaccinated individuals with comorbidities showed that for them, vaccination is vital because of the worse course of the disease and poor prognosis. Vaccination affects everyone differently depending on comorbidities or specific conditions. Clinical studies have shown that only some patients after kidney transplantation or chronic kidney disease undergoing hemodialysis developed a positive antibody response after two doses. The production of antibodies only after the third vaccination dose increased significantly [27,28]. It is often difficult for immunosuppressed patients who use immunosuppressive drugs and people who use high doses of steroids to achieve the same positive antibody levels as healthy individuals, as their immune system is weak [29]. Therefore, vaccinations are necessary to create sufficient protection. Much research is still being carried out and ongoing surveillance is underway, but it is already clear that individuals with chronic liver disease and those who have had a liver transplant do not produce enough antibodies after two doses, so a third dose of the vaccine is necessary [25]. In patients with autoimmune diseases, the drugs received and the nature of the autoimmune disease itself influence seroconversion. After the first vaccination, in the blood of patients, the anti-S antibodies were detected in approximately half (58.7%) of the participants, and, after receiving the full vaccination schedule, antibodies increased and were detected in the majority (85.1%) of participants [30]. It has been found that the production of fewer antibodies in vaccinated individuals with inflammatory intestinal disease is influenced by the use of some drugs. Therefore, it is very important not to delay the second vaccination dose and to get vaccinated on time [31]. A high body mass index and obesity also contribute to the formation of weaker immunity, negatively affect the cardiovascular system, and increase the risk of developing diabetes. Therefore, vaccinations are necessary for such people, although even after vaccination, due to lower immunity, there is a higher risk of infection [32].

Scientific studies showed that after contracting COVID-19 and receiving additional vaccinations, very strong immunity is formed that protects against reinfection. It is also stated that after vaccination, it is still possible to become infected with the SARS-CoV-2 virus, but vaccination prevents severe forms of the disease and bad outcomes. Therefore, it is very important to vaccinate regularly, especially people with comorbidities and specific conditions.

The evaluation of the immune response to vaccination in the long-term period of employees in a healthcare institution is very important, because these individuals work on the frontline and are exposed regularly to various variants of the virus and act as potential spreaders of the virus to potentially vulnerable people. Because of the variable exposure, the antibody response in healthcare workers can be variable and different from other people who were vaccinated against COVID-19.

The aim of the study was to evaluate the immune response to vaccination of healthcare frontline employees vaccinated against COVID-19.

## 2. Materials and Methods

### 2.1. Study Design and Participants

A quantitative study on the immune response to COVID-19 vaccination in frontline healthcare employees was conducted. The antibody titers of SARS-CoV-2 IgG (anti-RBD) were analyzed; a similar test for all participants was used. Anti-RBD are antibodies to the receptor binding domain of the S1 subunit of the spike protein of SARS-CoV-2. The antibody titers were determined by the neutralization method. The same protein variant was used to determine antibody titers throughout the study period. The reagents used were the SARS-CoV-2 IgG (anti-RBD) assay reagent kit. All subjects were routinely tested at regular intervals for COVID infection. COVID-19 was verified and documented by PCR testing. The strains with which subjects became infected at different times were identified.

The research was performed from December 2020 to September 2022, in Vilnius University Hospital Santaros Klinikos, (Lithuania) in Vilnius, the capital of the country. The study was conducted in one of the biggest Lithuanian hospitals providing the highest-level specialized personal health care services. This healthcare institution provides tertiary-level health care services. Tertiary level means specialized outpatient and inpatient personal health care services. There are 6300 employees, of whom 1663 are physicians, 2277 are nurses, 765 are nursing assistants, and 1595 are other employees in the hospital. The study was performed when the earliest vaccination against COVID-19 started, from 27 December 2020.

The first dose of Pfizer-BioNTech Comirnaty^®^ vaccine (manufacturer BioNTech Manufacturing GmbH, Pfizer Manufacturing Belgium NV) was used to vaccinate 1800 personal health care unit employees with a high risk of contracting COVID-19. Employees from the following frontline units were vaccinated: COVID-19 Units, Intensive Care Units, and Emergency Departments.

### 2.2. Inclusion and Exlusion Criteria

**Figure 1:**
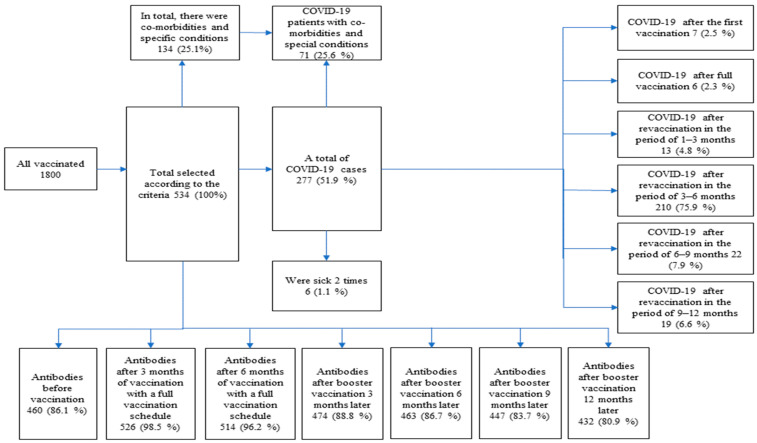
Flow chart of the study participants.

In order to obtain reliable statistical data and present the correct results and conclusions, the data used in the study were standardized according to the following selection criteria for healthcare workers who performed antibody tests against SARS-CoV-2. A total of 534 study participants were selected (Figure 1). Criteria for inclusion in the study group were:

- subjects who have signed biomedical research consent;
- subjects who did not have COVID-19 before vaccination or did not have laboratory evidence for the disease confirmed by PCR testing;
- employees vaccinated on 27–31 December 2020.

Exclusion criteria were:

- refusal to participate in a biomedical study;
- the employees who no longer worked at the hospital where this investigation was conducted;
- the employees who did not perform the antibody test at the specified time.

### 2.3. Measurement Tools

Adjusted biomedical research questionnaires were used to achieve the aim of the study. The two questionnaires consisted of items from questionnaires used in biomedical research at the hospital and were supplemented with data on the number of antibodies after vaccination with a full vaccination schedule and a booster dose. A person is considered to have received the full vaccination schedule if they have received two doses of the Comirnaty vaccine. The third dose of the vaccine is a booster dose.

Two biomedical survey questionnaires were used for the study. The first questionnaire was divided into four main parts. These were as follows:

- personal data (age, gender, height, weight);
- information about the respondent’s existing concomitant diseases or specific conditions that the person has at the time of vaccination;
- information about vaccination;
- the number of antibodies after vaccination according to the full vaccination schedule, and with the booster dose.

The first three parts are answered by the respondent, and the last part is filled in by the researcher using information from the database of the hospital. The second questionnaire was used after the participant had been diagnosed with COVID-19.

### 2.4. Bioethics

Written informed biomedical research consent was obtained and a patient consent form was signed by all subjects involved in the study. The data used in this study are aggregated data that have no personal identifying information that can be linked to the study participants. Patient consent was well preserved. The data we analyzed were de-identified.

All the study participants signed a patient consent form, and among other issues, they agreed that the data analyzed and the results obtained during the biomedical research can be published in scientific publications and/or presented at scientific conferences.

Permission from the general director of the institution (Vilnius University Hospital Santaros Klinikos) (No. SR-1483) was obtained to conduct the research. Approval from the Bioethics Committee at Klaipeda University (Protocol No.46Sv-VS-03) was obtained.

### 2.5. Statistical Analysis

Statistical data analysis was performed using SPSS 26 and Microsoft Excel 2023 program packages; the chi-square (χ^2^) criterion was applied. The difference in data was considered statistically significant when *p* < 0.05.

## 3. Results

### 3.1. Respondents

The average age of the healthcare workers was 45.4 ± 12.2 years. The youngest respondent was 21 years old and the oldest was 75 years old. The research participants were divided into five age groups: 21–30 years (15.4%), 31–40 years (19.7%), 41–50 years (26.8%), 51–60 years (26.2%), and 61 and over (12.0%). Of the 534 respondents who participated in the survey, 416 (77.9%) were female and 118 (22.1%) were male (Table 1).

Regarding the distribution of respondents according to body mass index, the calculation showed that the lowest body mass index (BMI) was 16.14, and the highest was 44.98. Most of the female respondents (187, 45.0%) were of normal weight, 13 (3.1%) were underweight, and 83 (20%) belonged to the I–III degree obesity groups. Most of the men (49, 41.5%) were overweight, 1 (0.8%) respondent was underweight, and 23 (19.9%) belonged to the I–II degree obesity groups (Table 2).

### 3.2. Distribution of Respondents Who Developed COVID-19 after Vaccination by Age, Gender, and Body Mass Index

The number of study participants who contracted COVID-19 after vaccination was compared according to the age groups. No statistically significant difference was found (*p* > 0.05) when comparing the frequency of illness after vaccination with COVID-19 between men and women, no statistically significant difference was observed. Out of all 277 participants who developed COVID-19 after vaccination, a third (100/36.1%) of participants were overweight, and 45 (16.2%) had grade I obesity. There was no significant difference observed (*p* > 0.05). After separately calculating the incidence of COVID-19 among participants with III-degree obesity, it was determined that five (83.3%) participants fell ill. This is statistically significant (*p* < 0.05) (Table 3).

### 3.3. Changes in the Number of SARS-CoV-2 IgG (Anti-RBD) Antibody Titers of Study Participants between Vaccinations

SARS-CoV-2 IgG (anti-RBD) antibodies were evaluated based on data from accredited laboratories. Antibodies (Ab) are considered positive when their serum levels exceed >50 AU/mL (AU—antibody units). The titer of SARS-CoV-2 IgG (anti-RBD) antibodies tested was divided into groups with different levels of antibodies (AU/mL): I: 1–49, II: 50–99, III: 100–499, IV: 500–999, V: 1000–4999, VI: 5000–9999, VII: 10,000–19,999, VIII: 20,000–29,999, and IX: 30,000–40,000.

#### 3.3.1. Antibodies to SARS-CoV-2 IgG (Anti-RBD) before Vaccination

Most participants had an antibody test before the first dose of the vaccine. Out of all 534 subjects, Ab were examined in 460 (86.1%) respondents. Among all the participants, the Ab test was positive in only one (0.2%) participant—61.5 AU/mL. The mean level of SARS-CoV-2 IgG (anti-RBD) antibodies in unvaccinated individuals was 2.9 ± 5.2 AU/mL. Antibody levels in almost all 459 (99.8%) participants were in group I (1–49). A total of 60 (11.2%) participants had an antibody level of 0.00 AU/mL (Table 4).

#### 3.3.2. SARS-CoV-2 IgG (Anti-RBD) Antibodies at 3 and 6 Months after Full Vaccination

In the study of 526 (98.5%) participants three months after full vaccination (after two doses), the observed mean level of SARS-CoV-2 IgG (anti-RBD) antibodies was 3879 ± 394 AU/mL. The lowest value was 137 and the highest was 39 972 AU/mL. The majority of participants (349, 66.3%) had antibodies which were distributed in the V antibodies group (4999–10,000). A total of 95 (18.1%) participants’ amount of antibodies belonged to the VI group (5000–9999).

Comparing the increases in SARS-CoV-2 IgG (anti-RBD) antibody levels in the different groups (I–VI) before vaccination and three months after vaccination did not show any significant differences (χ^2^ = 4.351; dƒ = 6; *p* > 0.05).

Six months after full vaccination, in 514 (96.3%) participants the mean level observed in the SARS-CoV-2 IgG (anti-RBD) antibody test was 1464 ± 2655 AU/mL. One (0.2%) participant had the lowest value, 58.0 AU/mL, and one (0.2%) participant had the highest value, 40,000 AU/mL. From the tested antibodies, 218 (40.8%) were distributed in the V (1000–4999), 168 (32.7%) in the IV (500–999), and 109 (21.2%) in the III (100–499) groups.

We compared antibody levels in 508 (95.1%) participants by groups at three and six months after full vaccination. Most antibodies showed a statistically significant decrease at six months (χ^2^ = 639.864; dƒ = 42; *p* < 0.05) (Table 4).

### 3.4. SARS-CoV-2 IgG (Anti-RBD) Antibodies after Booster Vaccination at 3, 6, 9, and 12 Months

Employees who received the first dose of the Comirnaty Pfizer vaccine in December 2020, and the second dose in January 2021, were administered a booster dose of the vaccine in September 2021. This means that all the subjects were given a booster dose 9 months after the first dose (in December 2020), and then the antibody levels were measured 3, 6, 9, and 12 months after all received the booster dose in September 2021.

**Figure 2:**
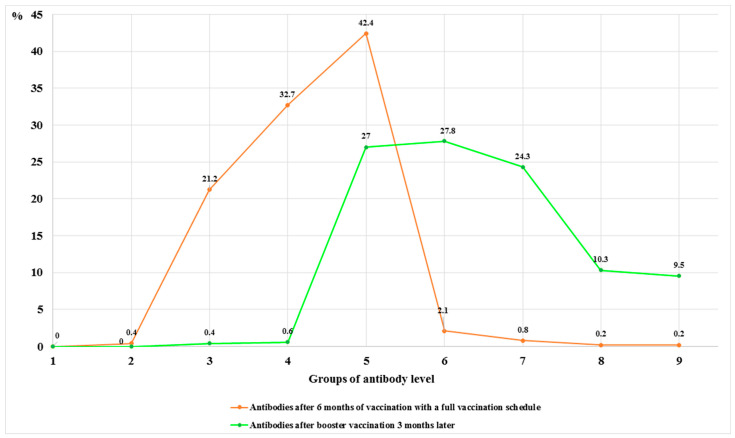
Change of antibody level six months after the full vaccination (two doses) and three months after booster vaccination.

Three months after revaccination, 474 (88.8%) participants had a mean SARS-CoV-2 IgG (anti-RBD) antibody titer dynamics of 12450.1 ± 10757.1 AU/mL. Two (0.4%) participants had the lowest value—222.5 AU/mL, and 45 (9.5%) participants had the highest value—40,000 AU/mL. Antibodies against SARS-CoV-2 were similarly distributed in groups V—128 (27.0%), VI—132 (27.8%), and VII—115 (24.3%). A comparison of the antibody distribution in the groups six months after full vaccination (two doses) and three months after booster vaccination showed a statistically significant increase in antibodies (χ^2^ = 639.864; dƒ = 42; *p* < 0.05) (Figure 2).

**Figure 3:**
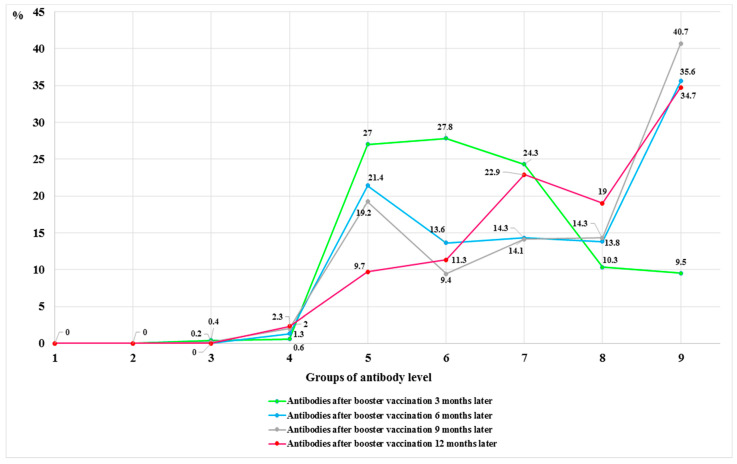
Change in the number of antibodies in the groups after the full vaccination (two doses) and after vaccination with a booster dose at 3, 6, 9, and 12 months.

We compared the change in antibody levels in the groups after vaccination with a booster dose at 3, 6, 9, and 12 months. In the majority of study participants (375, 70.2%), antibodies after revaccination at 3 months were almost equally distributed in groups V (1000–4999)—128 (27.0%), VI (5000–9999)—132 (27.8%), and VII (10,000–19,999)—115 (24.3%). After 6 months, antibody levels remained high and were distributed as follows: 99 (21.4%) in group V (1000–4999), 63 (13.6%) in group VI (5000–9999), 66 (14.3%) in group VII (10,000–19,999), 64 (13.8%) in group VIII (20,000–29,999), and even 165 (35.6%) in group IX (30,000–40,000). No significant change was observed after 9 months, with antibodies remaining in almost the same positions as after 6 months. After 12 months, the number of antibodies in group V decreased by more than half, with only 42 (9.7%) respondents remaining; the levels of antibodies in group VI only slightly changed, with a high percentage of antibodies remaining in high positions (high groups) (Figure 3).

### 3.5. Distribution of Study Participants Who Contracted COVID-19 after Vaccination

To determine the importance of antibodies and their influence on SARS-CoV-2 infection and disease occurrence, participants were asked if they had contracted COVID-19 after vaccination. All study participants who had a confirmed disease by PCR testing were investigated. Out of a total of 534 (100%) respondents, 277 (51.9%) participants fell ill with COVID-19 after vaccination, with 6 (1.1%) participants experiencing illness twice. The frequency of illnesses was estimated between vaccinations, distinguishing two stages of immunization: after receiving a full vaccination schedule (two doses) and after vaccination with a booster dose. In total, 277 (51.9%) participants fell ill after immunization. After the first vaccination, 7 (2.5%) participants had COVID-19, 6 (2.3%) after receiving the full vaccination schedule (two doses), and 13 (4.8%) participants had the disease 1–3 months after revaccination. After revaccination in the 3–6-month period, 210 (75.9%) participants fell ill, while after revaccination in the 6–9-month period, 22 (7.9%) participants fell ill, and after revaccination in the 9–12-month period, 19 (6.6%) participants contracted COVID-19 (Figure 1). The largest increase in the number of patients with COVID-19 occurred in the 3–6-month period after revaccination, with the majority (150, 54.2%) of patients falling into Ab groups V–VII (Table 5).

**Figure 4:**
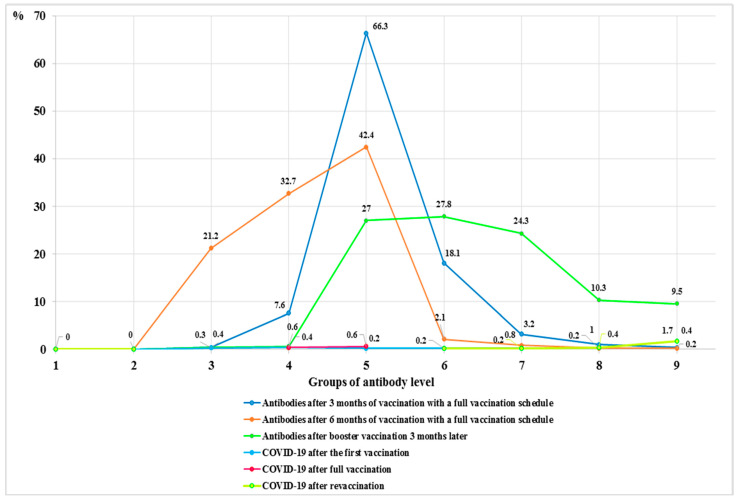
A comparison of the number of antibodies in the groups after full vaccination (two doses) between vaccinated patients with COVID-19 and vaccinated participants without COVID (during the Delta strain period).

When comparing the number of antibodies after full vaccination (two doses) between vaccinated individuals without COVID and vaccinated individuals who contracted COVID-19, no statistically significant change was observed. The number of patients after the first vaccination was divided between Ab groups I–IV and V–VI, while the number of patients after full vaccination was distributed between Ab groups IV–V. However, the level of antibodies after the booster dose was higher and distributed between Ab groups VI–IX (Figure 4).

We compared antibody groups between vaccinated participants with COVID-19 and vaccinated participants without COVID-19 at 3, 6, 9, and 12 months after the booster dose. The antibody levels of patients with COVID-19 were lower than those of non-diseased patients, and were distributed among groups V, VI, and VII (Table 4 and Table 5). When comparing vaccinated study participants without COVID-19 and those with COVID-19 infection, the change in antibodies after vaccination with the full vaccination schedule (two doses) and after vaccination with a booster dose was significantly lower in infected participants (χ^2^ = 98.370; dƒ = 25; *p* < 0.05).

We investigated the number of antibodies in diseased participants by group after the booster dose at 3, 6, 9, and 12 months. The lowest level was observed after 3 months after revaccination and was distributed between Ab groups V–VII. In those who were ill after 6 months after revaccination, the antibody titer remained high and distributed between the Ab groups VII–IX and remained similar at 9 and 12 months. The change in antibody titer between revaccination after 3 months and after revaccination after 12 months was determined to be statistically significant χ^2^ = 54.228; dƒ = 20; *p* < 0.05 (Table 5).

### 3.6. The Influence of Comorbidities and Specific Conditions on the Occurrence of COVID-19 and Changes in Antibody Levels

Respondents were asked about their current state of health at the time of vaccination and were asked to indicate any diseases or medical conditions listed in Questionnaire 1. Out of 534 respondents, 134 (25.1%) had comorbidities or specific conditions. Respondents with comorbidities and specific conditions were divided into those who developed COVID-19 after vaccination and those who did not. No statistically significant difference was found between the two groups (χ^2^ = 4.792; dƒ = 4; *p* > 0.05). Respondents who had comorbidities and specific conditions and were infected with the SARS-CoV-2 virus had a confirmed diagnosis of the disease. Among those with COVID-19, 45 (54.9%) respondents had primary arterial hypertension (PAH), 4 (40.0%) had type 2 CD, and 8 (66.7%) had asthma (Supplementary Materials Table S1).

Out of all the participants, 110 (20.6%) had one concomitant disease or specific condition, 24 (4.5%) had two or more, and 1 (0.2%) had five concomitant diseases or specific conditions. Among the 277 (100%) respondents who developed COVID-19 after vaccination, 71 (25.6%) had at least one comorbidity or specific condition, which accounted for more than half (52.9%) of all respondents with comorbidities. The remaining 63 (47.0%) respondents with comorbidities and specific conditions did not have the disease or did not have the disease confirmed by PCR testing (Supplementary Materials Table S2).

By choosing a period of 6 months after receiving the full vaccination schedule (two doses), when the Ab amount naturally begins to decline, antibody levels were compared between respondents with and without comorbidities and specific conditions. Although no statistically significant change was found (*p* > 0.05), Ab were distributed in lower antibody level groups (III–V) in all participants who had concomitant diseases or specific conditions. In group II, one patient (8.3%) had asthma, and two (20.0%) patients with type 2 CD were identified in the group VI (Supplementary Materials, Table S3).

By choosing a period of 3 months after receiving the booster dose, when Ab are at the highest level, Ab levels were compared between respondents with and without comorbidities and specific conditions. Although no statistically significant change was found (*p* > 0.05), Ab was distributed in higher antibody level groups in all participants who had concomitant diseases or specific conditions. In the lowest, group III, one (10.0%) patient had type 2 diabetes and one (1.4%) patient had primary arterial hypertension (Supplementary Materials Table S4).

## 4. Discussion

### 4.1. Main Findings

In our study, we found that after the country began to vaccinate healthcare workers, new cases of COVID-19 decreased significantly, and that vaccination against COVID-19 was an effective means of protection. Our results revealed that after receiving the full vaccination schedule, a sustained immune response was obtained, which was stable for up to 6 months; then, after receiving a booster dose, antibody levels remained high for another 12 months. However, even high levels of antibodies did not protect against the new strain of COVID, and there is a risk of being infected. We found that people with comorbidities and specific conditions had lower number of antibodies.

### 4.2. Interpretation of Results

Healthcare workers are definitely a priority group for vaccination against COVID-19 [8]. Therefore, healthcare workers were vaccinated first, in order to protect the vulnerable part of the population and frontline employees and to stop the spread of the virus and the spread of COVID-19. We analyzed the immune response and the titers of the obtained antibodies in this target group. Serology testing helped to identify past infection by quantifying the immune response. Individual immune responses are time dependent, which is reflected in antibody measurements [33].

The obtained results prove the benefits of vaccination after receiving a full vaccination scheme. A consistent and high titer immune response was created, so our research showed that vaccination and revaccination are the most effective means of stimulating the production of new antibodies and activating old antibodies. These data agree with the conclusions of other studies [20].

Based on the obtained results, we found that revaccination significantly increases the antibody titer, and the booster dose is very important to stop the spread of the COVID-19 epidemic. This supports the conclusions of other researches [11,12,13,14,15]. After a booster dose, the antibody titer can be significantly higher than after previous two doses [11,34,35]. However, the influence of the type of strain on the probability of infection became apparent. We found that the risk of infection depends on the type of strain circulating, and, during revaccination, existing antibodies are inactivated, so the chance of falling ill is reduced [20].

Our study revealed that revaccination is very important for the antibody titer change in patients with concomitant diseases and specific conditions, when only the third booster dose significantly increased the amount of antibodies; therefore, only after the third vaccination did the production of neutralizing antibodies start. This is especially important to prevent the spread of COVID-19. Other authors confirm this fact as well [27,29].

### 4.3. Limitations and Strengths

The study’s findings should be considered in the light of certain limitations. Not every participant in the study completed all of the scheduled antibody tests or arrived at specific scheduled times, which complicated the course of the study and might have compromised the accuracy of the results. In addition, study participants could hide/conceal the fact that they were ill, which could have changed the interpretation of the results. Despite the potential limitations, this study has also strong sides, including the fact that the monitoring of the immune response was carried out at different periods, so the evaluation of the period of the elimination of antibodies was carried out consistently. A clear increase in antibody titer was observed after vaccination with the full vaccination schedule and booster dose; thus, we demonstrated an evident positive association between vaccination and immune status. Most medical staff willingly agreed to participate in the biomedical study, and therefore followed the discipline and performed the antibody tests in a timely manner, which helped to collect optimal and reliable information. The study group was of various ages and with various comorbidities or conditions, which helped to reveal and evaluate the results of the study in a multifaceted manner.

### 4.4. Future Research

Future research on the immune response to the vaccination is important. It is recommended to repeat the monitoring of the change of antibody titers after a longer period (after 1–3 years) and evaluate how the immune response responds to new strains of COVID-19 and vaccination. In order to assess in which cases there is a higher probability of contracting COVID-19, it is recommended to compare the elimination time of antibodies in unvaccinated and vaccinated persons and to observe in which group the antibodies last longer. Later studies could be conducted in a larger population of people and also in other target groups and other work sectors.

In our study, the number of respondents with comorbidities and specific conditions among the participants was low. In order to assess the influence of comorbidities and specific conditions on the incidence of COVID-19, a greater number of participants with concomitant diseases should be investigated. New studies in healthcare institutions are necessary, conducting the research over a longer period of time and including as many clinically appropriate vaccinated and unvaccinated patients with COVID-19 as possible. Further studies on immune response and COVID-19 management are of great importance in order to control possible new pandemics.

## 5. Conclusions

The incidence of COVID-19 among frontline healthcare employees was reduced significantly after vaccination started in the healthcare sector. SARS-CoV-2 antibodies were detected in high-level antibody groups, and this level of antibodies was found to be effective in preventing COVID-19. Our results indicate that a sustained immune response was achieved after completing a full vaccination schedule, which remained stable for up to 6 months. After receiving a booster dose, antibody levels remained high for an additional 12 months. Although SARS-CoV-2 antibody levels decreased sharply after 6 months, even lower levels of antibodies provided protection against the Delta strain. The booster dose distributed the antibody titers in the high-level antibody groups, offering maximum protection at 12 months. However, even individuals with high AB titers were observed to contract COVID-19 after vaccination with a booster dose and 6 months in the presence of the Omicron strain. Unfortunately, high levels of antibodies did not provide protection against the new strain of COVID (the Omicron variant), posing a risk of infection.

When comparing the antibody titer of vaccinated participants without COVID-19 and those with COVID-19 infection, the change in antibodies after vaccination was significantly lower in infected participants. After assessing the influence of comorbidities and specific conditions on the dynamics of antibody titers, it was established that individuals with comorbidities and specific conditions had lower antibody levels. Therefore, the strategy of prioritizing individuals with comorbidities in the vaccination process was appropriate. It was found that patients with type 2 diabetes, asthma, and primary arterial hypertension need regular revaccination to generate a sufficient amount of antibodies to achieve the same level of protection as individuals without comorbidities and specific conditions. Research on the immune response to prevent COVID-19 and control new pandemics needs to be continued.

## Figures and Tables

**Table 1:** Study participants (by gender and age groups).

Gender		Age Groups					Total
		21–30 Years	31–40 Years	41–50 Years	51–60 Years	≥61	
Female	N	62	73	112	120	49	416
	%	14.9%	17.6%	26.9%	28.8%	11.8%	100%
Male	N	20	32	31	20	15	118
	%	16.9%	27.1%	26.3%	16.9%	12.7%	100%
Total	N	82	105	143	140	64	534
	%	15.4%	19.7%	26.8%	26.2%	12.0%	100%

**Table 2:** Study participants (by body mass index and gender).

**Gender**		**Body Mass Index (BMI) (kg/m^2^)**						**Total**
		**Underweight** BMI < 18.5	**Normal Weight** BMI 18.5 to 24.9	**Overweight** BMI 25.0 to 29.9	**I° Obesity** BMI 30.0 to 34.9	**II° Obesity** BMI 35.0 to 39.9	**III° Obesity** BMI ≥ 40.0	
Female	N	13	187	133	63	14	6	416
	%	3.10%	45.0%	32. 0%	15.10%	3.40%	1.40%	100%
Male	N	1	45	49	18	5	0	118
	%	0.80%	38.10%	41.50%	15.30%	4.20%	0.00%	100%
Total	N	14	232	182	81	19	6	534
	%	2.60%	43.4%	34.10%	15.20%	3.60%	1.10%	100%

**Table 3:** Distribution of respondents who developed or did not develop COVID-19 after vaccination by age, gender, and body mass index.

**With COVID-19**	**Gender**		**Age Groups**					**Total** **(N, %)**
	**Male**	**Female**	**21** **–30 Years**	**31** **–40 Years**	**41** **–50 Years**	**51** **–60 Years**	**≥61** **Years**	
	64 23.1%	213 76.9%	38 13.7%	59 21.3%	74 26.7%	81 29.2%	25 9.0%	277 100%
**Underweight** BMI < 18.5			3 42.9%	3 75.0%	0 0.0%	0 0.0%	1 100%	7 2.5%
**Normal weight** BMI 18.5 to 24.9			26 44.8%	32 54.2%	33 50.0%	17 44.7%	3 27.3%	111 40.1%
**Overweight** BMI 25.0 to 29.9			6 54.5%	18 62.1%	25 52.1%	38 65.5%	13 36.1%	100 36.2%
**I° Obesity** BMI 30.0 to 34.9			3 60.0%	5 45.5%	14 58.3%	18 60.0%	5 45.5%	45 16.2%
**II° Obesity** BMI 35.0 to 39.9			0 0.0%	1 50.0%	1 33.3%	5 55.6%	2 50.0%	9 3.2%
**III° Obesity** BMI ≥ 40.0			0 0.0%	0 0.0%	1 100%	3 75.0%	1 100%	5 1.8%
**No COVID-19**	**Gender**		**Age Groups**					**Total** **(N, %)**
	**Male**	**Female**	**21** **–30 Years**	**31** **–40 Years**	**41** **–50 Years**	**51** **–60 Years**	**≥61** **Years**	
	54 21.0%	203 79.0%	44 17.1%	46 17.9%	69 26.8%	59 23.0%	39 15.1%	257 100%
**Underweight** BMI < 18.5			4 57.1%	1 25.0%	1 100%	1 100%	0 0.0%	7 2.7%
**Normal weight** BMI 18.5 to 24.9			32 55.2%	27 45.8%	33 50.0%	21 55.3%	8 72.7%	121 47.1%
**Overweight** BMI 25.0 to 29.9			5 45.5%	11 37.9%	23 47.9%	20 34.5%	23 63.9%	82 31.9%
**I° Obesity** BMI 30.0 to 34.9			2 40.0%	6 54.5%	10 41.7%	12 40.0%	6 54.5%	36 14.0%
**II° Obesity** BMI 35.0 to 39.9			1 100%	1 50.0%	2 66.7%	4 44.4%	2 50.0%	10 3.9%
**III° Obesity** BMI ≥ 40.0			0 0.0%	0 0.0%	0 0.0%	1 25.0%	0 0.0%	1 0.4%
**Total**			82 15.4%	105 19.7%	143 26.8%	140 26.2%	64 12.0%	534 100%

**Table 4:** SARS-CoV-2 IgG (anti-RBD) antibodies after vaccination.

Groups	Antibody Levels before Vaccination		Antibodies after 3 Months of Vaccination with a Full Vaccination Schedule (Two Doses)		Antibodies after 6 Months of Vaccination with a Full Vaccination Schedule (Two Doses)		Antibodies after Booster Vaccination 3 Months Later		Antibodies after Booster Vaccination 6 Months Later		Antibodies after Booster Vaccination 9 Months Later		Antibodies after Booster Vaccination 12 Months Later	
	N	%	N	%	N	%	N	%	N	%	N	%	N	%
I (1–49)	459	99.8	0	0	0	0	0	0	0	0	0	0	0	0
II (50–99)	1	0.2	0	0	2	0.4	0	0	0	0	0	0	0	0
III (100–499)	0	0	18	3.4	109	21.2	2	0.4	0	0	1	0.2	0	0
IV (500–999)	0	0	40	7.6	168	32.7	3	0.6	6	1.3	9	2.0	10	2.3
V (1000–4999)	0	0	349	66.3	218	42.4	128	27.0	99	21.4	86	19.2	42	9.7
VI (5000–9999)	0	0	95	18.1	11	2.1	132	27.8	63	13.6	42	9.4	49	11.3
VII (10,000–19,999)	0	0	17	3.2	4	0.8	115	24.3	66	14.3	63	14.1	99	22.9
VIII (20,000–29,999)	0	0	5	1.0	1	0.2	49	10.3	64	13.8	64	14.3	82	19.0
IX (30,000–40,000)	0	0	2	0.4	1	0.2	45	9.5	165	35.6	182	40.7	150	34.7
Total	460	100	526	100	514	100	474	100	463	100	447	100	432	100

**Table 5:** Changes in the number of antibodies in groups 3, 6, 9, and 12 months after the booster dose.

Groups	Distribution of Antibodies in the Post-Vaccination Patients by Ab Group (N, %)					
	COVID-19 after the First Vaccination	COVID-19 after Full Vaccination	COVID-19 after Revaccination in a Period of 1–3 Months	COVID-19 after Revaccination in a Period of 3–6 Months	COVID-19 after Revaccination in a Period of 6–9 Months	COVID-19 after Revaccination in a Period of 9–12 Months
	Antibodies after booster vaccination **3 months later**					
III	0 (0.0%)	0 (0.0%)	0 (0.0%)	1 (0.5%)	0 (0.0%)	0 (0.0%)
IV	1 (50.0%)	0 (0.0%)	0 (0.0%)	1 (0.5%)	0 (0.0%)	0 (0.0%)
V	1 (50.0%)	0 (0.0%)	0 (0.0%)	48 (25.7%)	4 (22.2%)	9 (47.4%)
VI	0 (0.0%)	1 (33.3%)	1 (8.3%)	52 (27.8%)	6 (33.3%)	4 (21.1%)
VII	0 (0.0%)	0 (0.0%)	1 (8.3%)	47 (25.1%)	5 (27.8%)	2 (10.5%)
VIII	0 (0.0%)	0 (0.0%)	2 (16.7%)	22 (11.8%)	3 (16.7%)	2 (10.5%)
IX	0 (0.0%)	2 (66.7%)	8 (66.7)%	16 (8.6%)	0 (0.0%)	2 (10.5%)
**Total**	2 (100%)	3 (100%)	12 (100%)	187 (100%)	18 (100%)	19 (100%)
	Antibodies after booster vaccination **6 months later**					
IV	0 (0.0%)	0 (0.0%)	0 (0.0%)	1(0.5%)	0 (0.0%)	1 (5.3%)
V	1 (25.0%)	0 (0.0%)	0 (0.0%)	11 (5.9%)	9 (47.4%)	10 (52.6%)
VI	1 (25.0%)	0 (0.0%)	1 (7.7%)	10 (5.4%)	4 (21.1%)	4 (21.1%)
VII	2 (50.0%)	1 (20.0%)	2 (15.4%)	23 (12.4%)	3 (15.8%)	3 (15.8%)
VIII	0 (0.0%)	2 (40.0%)	2 (15.4%)	40 (21.5%)	1 (5.3%)	1 (5.3%)
IX	0 (0.0%)	2 (40.0%)	8 (61.5%)	101 (54.3%)	2 (10.5%)	0 (0.0%)
**Total**	4 (100%)	5 (100%)	13 (100%)	186 (100%)	19 (100%)	19 (100%)
	Antibodies after booster vaccination **9 months later**					
III	0 (0.0%)	0 (0.0%)	0 (0.0%)	0 (0.0%)	0 (0.0%)	1 (5.9%)
IV	0 (0.0%)	0 (0.0%)	0 (0.0%)	0 (0.0%)	0 (0.0%)	4 (23.5%)
V	2 (40.0%)	0 (0.0%)	0 (0.0%)	5 (2.7%)	1 (5.9%)	9 (52.9%)
VI	2 (40.0%)	0 (0.0%)	3 (27.3%)	7 (3.8%)	1 (5.9%)	2 (11.8%)
VII	0 (0.0%)	1 (100%)	1 (9.1%)	30 (16.3%)	1 (5.9%)	0 (0.0%)
VIII	1 (20.0%)	0 (0.0%)	4 (36.4%)	37 20.1%)	2 (11.8%)	1 (5.9%)
IX	0 (0.0%)	0 (0.0%)	3 (27.3%)	105 (57.1%)	12 (70.6%)	0 (0.0%)
**Total**	5 (100%)	1 (100%)	11 (100%)	184 (100%)	17 (100%)	17 (100%)
	Antibodies after booster vaccination **12 months later**					
V	1 (16.7%)	0 (0.0%)	1 (7.7%)	4 (2.3%)	0 (0.0%)	0 (0.0%)
VI	4 (66.7%)	0 (0.0%)	2 (15.4%)	13 (7.3%)	2 (11.8%)	1 (5.9%)
VII	1 (16.7%)	2 (100%)	4 (30.8%)	45 (25.4%)	2 (11.8%)	1 (5.9%)
VIII	0 (0.0%)	0 (0.0%)	4 (30.8%)	49 (27.7%)	4 (23.5%)	2 (11.8%)
IX	0 (0.0%)	0 (0.0%)	2 (15.4%)	66 (37.3%)	9 (52.9%)	13 (76.5%)
**Total**	6 (100%)	2 (100%)	13 (100%)	177 (100%)	17 (100%)	17 (100%)

## Data Availability

Restrictions apply to the availability of these data. The data was obtained from a third party and are available with the permission of the third party.

## Supplementary Materials

The following supporting information can be downloaded at: https://www.mdpi.com/article/10.3390/vaccines12020199/s1, Table S1: Distribution of respondents according to comorbidities and specific conditions; Table S2: Study participants with comorbidities and specific conditions who developed COVID-19; Table S3: Participants with comorbidities and specific conditions and level of antibodies after 6 months of receiving the vaccination with full vaccination schedule (2 doses); Table S4: Participants with comorbidities and specific conditions, and the level of antibodies after 3 months of receiving the vaccination with booster dose.
