# Supplementary material for: Immune Response to COVID-19 Vaccination in Frontline Healthcare Workers
Source: Vaccines (Basel). 2024 Feb 15;12(2):199. doi: 10.3390/vaccines12020199 (PMC10891992; doi:10.3390/vaccines12020199)
Supplement: Supplementary file 1 [file vaccines-12-00199-s001.zip › vaccines-2822909-supplementary.pdf]

Supplementary Materials

Table S1. Distribution of respondents according to comorbidities and specific conditions.

**Comorbidities and specific conditions**	**Distribution of respondents according to comorbidities and specific conditions**		**Comparison of the number of respondents with comorbidities and specific conditions who had COVID-19 after vaccination**	
	**N**	**%**	**N**	**%**
Oncological disease	9	5.3	3	33.3
Autoimmune disease	32	18.7	15	46.9
Asthma	12	7.0	8	66.7
Chronic kidney disease (CKD)	3	1.8	0	0.0
Primary arterial hypertension (PAH)	82	48.0	45	54.9
Chemotherapy	3	1.8	0	0.0
Long-term treatment with glucocorticoids	1	0.6	0	0.0
Oncohematological disease	1	0.6	1	100
Type 2 CD	10	5.8	4	40.0
Chronic obstructive pulmonary disease (COPD)	3	1.8	0	0.0
Heart failure	6	3.5	3	50.0
Pregnancy	4	2.3	2	50.0
Biological therapy	3	1.8	2	66.7
Posttransplant immunosuppression	2	1.2	0	0.0
**Total**	171	100	83	48.5

Table S2. Study participants with comorbidities and specific conditions who developed COVID-19.

**Number of comorbidities and specific conditions**	**Number of study participants**	**Detected SARS-CoV-2**	**Not-detected SARS-CoV-2**
0	400 (74.9%)	206 (74.4%)	194 (75.5%)
1	110 (20.6%)	62 (22.4%)	48 (18.7%)
2	16 (3.0%)	5 (1.8%)	11 (4.2%)
3	7 (1.3%)	3 (1.0%)	4 (1.6%)
5	1 (0.2%)	1 (0.4%)	0 (0%)
Total	534 (100%)	277 (100%)	257 (100%)

Table S3. Participants with comorbidities and specific conditions and level of antibodies after 6 months of receiving the vaccination with full vaccination schedule (two doses).

	**Ab groups**									**Total**
	I	II	III	IV	V	VI	VII	VIII	IX	
	1–49	50–99	100–499	500–999	1000–4999	5000–9999	10 000–19 999	20 000–29 999	30 000–40 000	
**Total number of participants, who came for Ab investigation**										
	0	2	109	168	218	11	4	1	1	514

	0.0 %	0.4%	21.2%	32.7%	42.4%	2.1%	0.8%	0.2%	0.2%	100%
**Number of participants with comorbidities and specific conditions**										
Primary arterial hypertension (PAH)	0<br>0.0%	0<br>0.0%	28<br>34.6%	31<br>38.3%	22<br>27.2%	0<br>0.0%	0<br>0.0%	0<br>0.0%	0<br>0.0%	81<br>100%
Heart failure	0<br>0.0%	0<br>0.0%	4<br>66.7%	1<br>16.7%	1<br>16.7%	0<br>0.0%	0<br>0.0%	0<br>0.0%	0<br>0.0%	6<br>100%
Oncological disease	0<br>0.0%	0<br>0.0%	2<br>22.2%	4<br>44.4%	3<br>33.3%	0<br>0.0%	0<br>0.0%	0<br>0.0%	0<br>0.0%	9<br>100%
Oncohematological disease	0<br>0.0%	0<br>0.0%	0<br>0.0%	0<br>0.0%	1<br>100%	0<br>0.0%	0<br>0.0%	0<br>0.0%	0<br>0.0%	1<br>100%
Autoimmune disease	0<br>0.0%	0<br>0.0%	9<br>30.0%	9<br>30.0%	12<br>40.0%	0<br>0.0%	0<br>0.0%	0<br>0.0%	0<br>0.0%	30<br>100%
Type 2 CD	0<br>0.0 %	0<br>0.0%	6<br>60.0%	2<br>20.0%	0<br>0.0%	2<br>20.0%	0<br>0.0%	0<br>0.0%	0<br>0.0%	10<br>100%
Asthma	0<br>0.0%	1<br>8.3%	0<br>0.0%	1<br>8.3%	10<br>83.3%	0<br>0.0%	0<br>0.0%	0<br>0.0%	0<br>0.0%	12<br>100%
Chronic obstructive pulmonary disease (COPD)	0<br>0.0%	0<br>0.0%	2<br>66.7%	1<br>33.3%	0<br>0.0%	0<br>0.0%	0<br>0.0%	0<br>0.0%	0<br>0.0%	3<br>100%
Chronic kidney disease (CKD)	0<br>0.0%	0<br>0.0%	2<br>66.7%	0<br>0.0%	1<br>33.3%	0<br>0.0%	0<br>0.0%	0<br>0.0%	0<br>0.0%	3<br>100%
Pregnancy	0<br>0.0%	0<br>0.0%	0<br>0.0%	0<br>0.0%	4<br>100%	0<br>0.0%	0<br>0.0%	0<br>0.0%	0<br>0.0%	4<br>100%
Biological therapy	0<br>0.0%	0<br>0.0%	0<br>0.0%	2<br>66.7%	1<br>33.3%	0<br>0.0%	0<br>0.0%	0<br>0.0%	0<br>0.0%	3<br>100%
Long-term treatment with glucocorticoids	0<br>0.0%	0<br>0.0%	0<br>0.0%	1<br>100%	0<br>0.0%	0<br>0.0%	0<br>0.0%	0<br>0.0%	0<br>0.0%	1<br>100%
Posttransplant immunosuppression	0<br>0.0%	0<br>0.0%	1<br>50.0%	1<br>50.0%	0<br>0.0%	0<br>0.0%	0<br>0.0%	0<br>0.0%	0<br>0.0%	2
Total	0<br>0.0%	1<br>0.6%	54<br>32.7%	53<br>32.1%	55<br>33.3%	2<br>1.2%	0	0	0	165<br>100%

Table S4. Participants with comorbidities and specific conditions, and the level of antibodies after 3 months of receiving the vaccination with booster dose.

	**Ab groups**									**Total**
	I	II	III	IV	V	VI	VII	VIII	IX	
	1–49	50–99	100–499	500–999	1000–4999	5000–9999	10 000–19 999	20 000–29 999	30 000–40 000	
**Total number of research participants, who came for Ab investigation**										
	0<br>0.0%	0<br>0.0%	2<br>0.4%	3<br>0.6%	128<br>27.0%	132<br>27.8%	115<br>24.3%	49<br>10.3%	45<br>9.5%	474<br>100%
**Number of participants with comorbidities and specific conditions**										
Primary arterial hypertension (PAH)	0<br>0.0%	0<br>0.0%	1<br>1.4%	0<br>0.0%	23<br>32.4%	16<br>22.5%	15<br>21.1%	10<br>14.1%	6<br>8.5%	71<br>100%
Heart failure	0<br>0.0%	0<br>0.0%	0<br>0.0%	0<br>0.0%	2<br>40.0%	2<br>40.0%	0<br>0.0%	1<br>20.0%	0<br>0.0%	5<br>100%
Oncological disease	0<br>0.0%	0<br>0.0%	0<br>0.0%	0<br>0.0%	3<br>33.3%	1<br>11.1%	3<br>33.3%	0<br>0.0%	2<br>22.2%	9<br>100%
Oncohematological disease	0<br>0.0%	0<br>0.0%	0<br>0.0%	0<br>0.0%	0<br>0.0%	0<br>0.0%	1<br>100%	0<br>0.0%	0<br>0.0%	1<br>100%
Autoimmune disease	0	0	0	0	11	8	6	1	1	27

	0.0%	0.0%	0.0%	0.0%	40.7%	29.6%	22.2%	3.7%	3.7%	100%
Type 2 diabetes	0<br>0.0%	0<br>0.0%	1<br>10.0%	0<br>0.0%	5<br>50.0%	1<br>10.0%	1<br>10.0%	1<br>10.0%	1<br>10.0%	10<br>100%
Asthma	0<br>0.0%	0<br>0.0%	0<br>0.0%	0<br>0.0%	3<br>25.0%	3<br>25.0%	3<br>25.0%	2<br>16.7%	1<br>8.3%	12<br>100%
Chronic obstructive pulmonary disease (COPD)	0<br>0.0%	0<br>0.0%	0<br>0.0%	0<br>0.0%	1<br>33.3%	1<br>33.3%	1<br>33.3%	0<br>0.0%	0<br>0.0%	3<br>100%
Chronic kidney disease (CKD)	0<br>0.0%	0<br>0.0%	0<br>0.0%	0<br>0.0%	1<br>33.3%	0<br>0.0%	1<br>33.3%	0<br>0.0%	1<br>33.3%	3<br>100%
Pregnancy	0<br>0.0%	0<br>0.0%	0<br>0.0%	0<br>0.0%	1<br>25.0%	1<br>25.0%	2<br>50.0%	0<br>0.0%	0<br>0.0%	4<br>100%
Biological therapy	0<br>0.0%	0<br>0.0%	0<br>0.0%	0<br>0.0%	1<br>50.0%	1<br>50.0%	0<br>0.0%	0<br>0.0%	0<br>0.0%	2<br>100%
Long-term treatment with glucocorticoids	0<br>0.0%	0<br>0.0%	0<br>0.0%	0<br>0.0%	1<br>100%	0<br>0.0%	0<br>0.0%	0<br>0.0%	0<br>0.0%	1<br>100%
Posttransplant immunosuppression	0<br>0.0%	0<br>0.0	0<br>0.0%	0<br>0.0%	2<br>100%	0<br>0.0%	0<br>0.0%	0<br>0.0%	0<br>0.0%	2<br>100%
Total	0<br>0.0%	0<br>0.0%	2<br>1.3%	0<br>0.0%	54<br>36.0%	34<br>22.7%	33<br>22.0%	15<br>10.0%	12<br>8.0%	150<br>100%
